# Flies Get a Head Start on Meiosis

**DOI:** 10.1371/journal.pgen.1004051

**Published:** 2013-12-19

**Authors:** Cori K. Cahoon, R. Scott Hawley

**Affiliations:** 1 Stowers Institute for Medical Research, Kansas City, Missouri, United States of America; 2 Department of Molecular and Integrative Physiology, University of Kansas Medical Center, Kansas City, Kansas, United States of America; The University of North Carolina at Chapel Hill, United States of America

Few distinctions in biology are as clearly drawn as the one between mitosis and meiosis. The function of mitosis is to produce two identical daughter cells, while the purpose of the first division of meiosis is to ensure the segregation of homologous chromosomes, and the second division is to create haploid gametes. These meiotic segregations usually rely on meiosis-specific processes such as the induction of preprogramed double-strand breaks, homolog pairing, synaptonemal complex (SC) formation, synapsis, and recombination between homologs to form crossovers. While not all meiotic systems function identically, enough commonalities exist to allow us to draw clear distinctions between meiosis and mitosis. Mitotic cells simply do not build SC, and the process of full-length homolog pairing is almost always a prerogative of cells entering meiosis, not mitosis.

Vexingly for those of us who like our distinctions made clearly, all this has changed, at least in Drosophila, as a result of two papers in this issue of *PLOS Genetics* [1], [2]. These authors show that although homologs are neither paired nor synapsed in germline stem cells (GSCs) of Drosophila females, both homolog pairing and the accumulation of SC components in pericentromeric regions initiate during the five mitotic divisions preceding meiosis. While the mechanisms and consequences of these interactions remain to be elucidated, it is clear that pairing and synapsis begin earlier than previously imagined—indeed, they initiate during the premeiotic *mitotic* divisions.

## Models of Meiotic Pairing

Meiotic chromosome pairing in most organisms is thought to involve the formation of double-strand breaks and a subsequent single-stranded DNA homolog search, causing homologs to associate. This association is facilitated by the formation of the SC, a proteinaceous structure between homologs that is required for proper chromosome segregation. More recently, noncoding RNAs have been discovered that assist in the homolog search process [3], suggesting that other mechanisms may also exist to facilitate pairing.

However, the study of meiotic pairing in Drosophila has been challenging for several reasons. First, it has been difficult to cytologically observe chromosome pairing in fixed images, and no imaging techniques currently exist to adequately follow the pairing process live. Moreover, there are no known mutants that specifically affect the pairing process in flies. Another impediment lies in the fact that in dipterans, such as *Drosophila melanogaster*, homologs undergo an early pairing event near the end of syncytial blastoderm development [4], [5]. This pairing process continues throughout development and was previously thought to occur in all tissues, including the germline [6].

The existence of ubiquitous pairing has led to the (far too popular) hypothesis that the pairing established early in Drosophila embryogenesis is maintained in the germline, and that meiotic homolog pairing is therefore simply an extension of this somatic pairing [7]. However, Joyce et al. [2] clearly demonstrate that homologous chromosomes are *not* paired in either GSCs or their progenitors. Furthermore, homologous chromosome pairing occurs progressively through the early, pre-meiotic divisions in the germline [2] and is facilitated by the association of SC components near the centromeres [1]. The localization of these SC components to pericentromeric regions might reflect either the formation of mature SC (as seems most likely—see below) or the accumulation of SC components. Only much higher resolution microscopy can distinguish between these two alternatives, but for the sake of brevity we will simply use the term SC to describe these structures. To the best of our knowledge, this is the first demonstration of SC in mitotic cells of any organism and the first evidence that meiotic pairing in Drosophila is not the consequence of the pairing established in early embryogenesis.

## Drosophila Female Meiosis: How It Really Works

**Figure 1:**
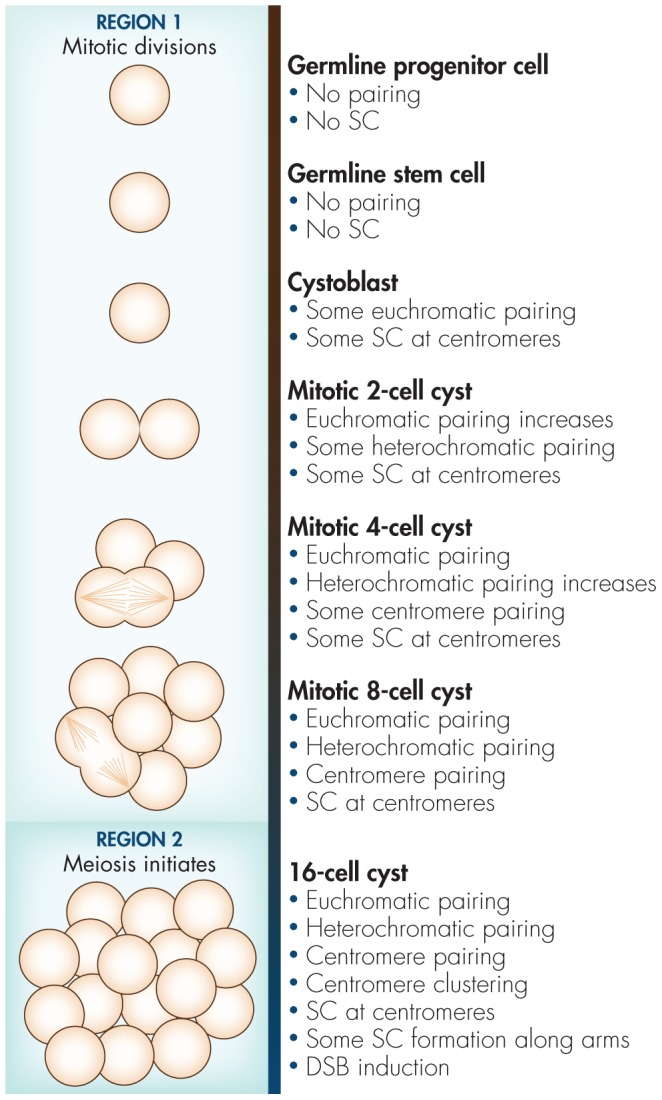
Diagram of events in premeiotic germline with respect to autosome pairing, centromere clustering, and SC formation based on the results from Joyce et al. [2] and Christophorou et al. [1]. (We thank Angela Seat for figure and editorial assistance.)

Female Drosophila meiosis occurs in the germarium of the ovariole, which is subdivided into three regions based on cytological observations of cell morphology [8]. At the anterior tip of the germarium, GSCs divide asymmetrically to produce a cystoblast that undergoes four consecutive mitotic divisions with incomplete cytokinesis, resulting in a cyst of 16 interconnected cells (Figure 1). These mitotically dividing cysts define region 1 of the germarium. After region 1, the 16-cell cyst stops dividing mitotically and starts meiosis in region 2.

Using a novel technique to mark specific chromosomes [9], Joyce et al. [2] report that GSCs and the germline progenitor cells from which GSCs arise, in fact, lack somatic pairing for both euchromatic and heterochromatic regions. Interestingly, there is an exception to the lack of somatic pairing in GSCs: The 359-base pair satellite sequence of the *X* heterochromatin—which borders the rDNA genes—is paired in 80% of GSCs [2]. The ability to pair *X* heterochromatin in virtually any cell type may well reflect the fact that *X* chromosomes carry the 18S and 28S rDNA clusters and thus share a common nucleolus. However, for the two autosomes tested, Joyce et al. [2] and Christophorou et al. [1] show that pairing progressively increases through the mitotic region as the cyst matures.

It is possible that germline progenitor cells and GSCs contain a block to the somatic pairing process that is alleviated as cystoblasts begin mitoses in region 1 (Figure 1), allowing for somatic pairing to be reestablished. However, this hypothesis fails to explain the surprising observation by Christophorou et al. [1] that SC components are associated near centromeres in the mitotically dividing cells in region 1. Meiotic proteins are not required for somatic chromosome pairing, and SC-defective mutants exhibit no homolog pairing defects in somatic cells [10]. Therefore, it seems likely that the pairing observed in the mitotic region of the germarium is in fact a meiotic process that differs from somatic cell pairing.

## Centromere Pairing in Mitosis Precedes Centromere Clustering in Meiosis

Does the observation of SC in mitotically dividing cells reflect the pre-loading of SC that will assemble into mature SC as meiosis initiates in region 2, or does it reflect the existence of mature, structurally normal SC? And perhaps more importantly, does this mitotic SC play a role in mediating homologous centromere pairing? The meiosis-specific protein components of the Drosophila SC are the lateral element proteins Ord [11] and C(2)M [12], which lie along the chromosome axes, the transverse filament protein C(3)G [13], which spans the width between two homologs, and the central element protein Cona [14], which helps stabilize the region between homologs.

Christophorou et al. used mutants of these SC components to show that Cona and C(3)G promote centromere association during mitosis in region 1 [1]. Most critically, they also show that mutational ablation of C(3)G and Cona impairs the progression of centromeric pairing in region 1 and centromere clustering (the association of paired centromeres) [1]. These observations support the view that the SC-like structures observed by these authors may well be functional SC. Christophorou et al. further demonstrate that mitotic pairing of homologous centromeres in region 1 occurs before paired homologous centromeres cluster into one or two masses in early region 2 [1]. However, the aggregation of paired centromeres into one or two major foci does not occur until the canonical initiation of meiosis in early region 2, which is consistent with prior observations by Takeo et al. and Tanneti et al. [15], [16]. Recently, Unhavaithaya and Orr-Weaver have shown that the centromere clustering process requires the function of two centromere-associated proteins, CAL1 (centromere nucleosome loader) and CENP-C (component of the centromere associated network, which is required for assembly of kinetochores in mitosis) [17].

It is unclear how the SC or its components function to help pair homologous centromeres in region 1. While SC mutants are known to disrupt centromere clustering in early region 2 proocytes, it is intriguing that although centromere pairing in region 1 occurs more slowly in these mutants, it is not abolished [1], suggesting that another SC-independent pathway of centromeric pairing exists. Although it remains to be seen, meiotic pairing in flies may not be that different from yeast and worms, where there is evidence that diffusion is a force that drives pairing [18], [19]. Nevertheless, the evidence that meiotic SC components are both present *and necessary* for efficient homologous centromere pairing in mitotic cells in region 1 further erodes the notion that meiotic pairing is simply an extension of somatic pairing.

## What Now?

These results create a puzzling situation. How can a cell undergo mitosis with SC present near homologous centromeres? Moreover, as Christophorou et al. [1] observed, SC is already present in 4-cell cysts, which must undergo two additional, incomplete, mitotic divisions (Figure 1). If the SC present near homologous centromeres associates with only one sister chromatid from each homolog, it is possible that chromosomes could orient in such a way that the two sisters lacking SC could segregate to one pole, while the SC-associated sisters segregate to the other. However, this raises the question: How does a replication fork move through SC-associated centromeres? Additionally, if SC *is* built between the two pairs of sisters, then the only way a cell could mitotically divide would be to disassemble the SC at every division. Why would a cell build centromeric SC, only to dismantle and rebuild it after every division? It is becoming increasingly apparent that pericentromeric SC differs from the SC along chromosome arms [20], [21]. Perhaps this difference is key to understanding how and why, in Drosophila, pre-meiotic cells can undergo rapid mitotic divisions while associated with centromeric SC.
